# How to create value with unobtrusive monitoring technology in home-based dementia care: a multimethod study among key stakeholders

**DOI:** 10.1186/s12877-022-03550-1

**Published:** 2022-11-30

**Authors:** Christian Wrede, Annemarie Braakman-Jansen, Lisette van Gemert-Pijnen

**Affiliations:** Centre for eHealth and Wellbeing Research, Department of Psychology, Health and Technology, University of Twente, Drienerlolaan 5, Enschede, 7522 NB The Netherlands

**Keywords:** Unobtrusive monitoring technology, Assistive technology, Dementia, Home-based care, Aging in place, Value proposition design, Implementation

## Abstract

**Background:**

There is a growing interest to support extended independent living of people with dementia (PwD) via unobtrusive monitoring (UM) technologies which allow caregivers to remotely monitor lifestyle, health, and safety of PwD. However, these solutions will only be viable if developers obtain a clear picture of how to create value for all relevant stakeholders involved and achieve successful implementation. The aim of this study was therefore to explore the value proposition of UM technology in home-based dementia care and preconditions for successful implementation from a multi-stakeholder perspective.

**Methods:**

We conducted an expert-informed survey among potential stakeholders (*n* = 25) to identify key stakeholders for UM technology in home-based dementia care. Subsequently, focus groups and semi-structured interviews were conducted among 5 key stakeholder groups (*n* = 24) including informal caregivers (*n* = 5), home care professionals (*n* = 5), PwD (*n* = 4), directors and managers within home care (*n* = 4), and policy advisors within the aged care and health insurance sector (*n* = 6). The sessions addressed the value proposition- and business model canvas and were analyzed using thematic analysis.

**Results:**

Stakeholders agreed that UM technology should provide gains such as objective surveillance, timely interventions, and prevention of unnecessary control visits, whereas pains mainly included information overload, unplannable care due to real-time monitoring, and less human interaction. The overall design-oriented need referred to clear situation classifications including urgent care (fall- and wandering detection), non-urgent care (deviations in eating, drinking, sleeping), and future care (risk predictions). Most important preconditions for successful implementation of UM technology included inter-organizational collaboration, a shared vision on re-shaping existing care processes, integrated care ICT infrastructures, clear eligibility criteria for end-users, and flexible care reimbursement systems.

**Conclusions:**

Our findings can guide the value-driven development and implementation of UM technology for home-based dementia care. Stakeholder values were mostly aligned, although stakeholders all had their own perspective on what UM technology should accomplish. Besides, our study highlights the complexity of implementing novel UM technology in home-based dementia care. To achieve successful implementation, organizational and financial preconditions, as well as digital data exchange between home care organizations, will be important.

**Supplementary Information:**

The online version contains supplementary material available at 10.1186/s12877-022-03550-1.

## Background

Given the projected trends in population aging, the global prevalence of dementia is expected to almost triple by the year 2050 [1]. To reduce the tension between an increasing demand for care and the growing shortage in residential care capacity [2] people with dementia (PwD) will, as far as possible, be encouraged to live at home for longer [3, 4]. While most PwD prefer to live independently for longer, this typically places more pressure on their (in) formal care network [5]. Caring for community-dwelling PwD can be challenging and increases the risk of physical and mental health issues [6, 7]. The strain on (in)formal caregivers of PwD has furthermore intensified in the light of the Covid-19 restrictions [8, 9]. Decreased access to non-domestic support services, such as day care and respite care, has been causing greater reliance on home-based care as an important pillar of the healthcare system [10].

The increasing need for support of caregivers and PwD in the home environment calls for innovative solutions, including those from the field of health technology [11]. In the Netherlands, the National Dementia Strategy 2021–2030 [12] encourages the advancement of health technologies to support PwD and their caregivers. One specific form of technology that has been gaining growing interest is in-home monitoring technology. These technologies enable continuous monitoring of daily functioning, lifestyle, health, and safety of PwD, possibly allowing a greater sense of control among (in)formal caregivers when managing care demands, which could help to delay the institutionalization of PwD [13–15].

Different generations of in-home monitoring technologies exist. In this study, we focus on the most novel generation under development [16–20] which uses unobtrusive sensing (such as Wifi-CSI or radar) and artificial intelligence (AI) to model human activity. These unobtrusive monitoring (UM) technologies might outperform earlier generations of monitoring devices in several ways: First, UM technologies do not require any (in)direct body contact or being in line of sight (LOS), making them less prone to privacy and feasibility issues as compared to wearables or cameras [16, 21]. Second, UM technologies have the potential to capture continuous dynamic human activity as opposed to systems using motion-activated sensors attached to home equipment [21]. Additionally, AI-driven UM technologies could help caregivers of PwD to detect small but relevant health status changes over time, and not only detect but also predict falls and other adverse health events [22], thereby offering unique opportunities for person-centered care.

However, despite their potential and position within recent policy, research on intelligent UM technologies is lacking knowledge on how exactly to create value for home-based dementia care and achieve successful implementation. Earlier research has largely focused on investigating either technical system possibilities [23–27] or the needs of healthy older adults and their caregivers towards earlier generations of monitoring technologies [22, 28–30]. Few studies exist that explored the needs towards in-home monitoring technology intended to support home-based dementia care [13, 31, 32]. However, those studies have limited their focus to the perspective of end-users, rather than forming a more complete understanding of the perspectives of a broader range of stakeholders who might affect or be affected by the technology.

When developing novel UM technology to support home-based dementia care, the aforementioned knowledge gaps make it difficult to achieve a good fit between technology, stakeholders, and use context which increases the chance of non-adoption and unsuccessful implementation. Previous work highlights that taking a business modeling perspective in developing and implementing health technologies can improve the chance of achieving a fit between technology, stakeholders, and context [33–35]. The well-established business model canvas (BMC) of Osterwalder et al. [36] introduces research activities before the start of the actual technical design that can help to inform bottom-up value-based development and identify preconditions for sustainable implementation in practice [35]. Defining the value proposition of a technology for different stakeholder profiles takes a central role in this approach. This goes beyond the technology’s added value for the end-user(s) but also includes how the technology matches the technological, organizational, and economic needs of all relevant stakeholders [33, 36, 37]. Although the business modeling perspective has been integrated into widely-adopted health technology research frameworks such as the CeHRes Roadmap [37], it is still under-used in development and implementation of technology to support dementia care. This might be attributed to the fact that business modeling originates from a commercial setting, however, it can also be applied for any non-profit organization involved in the development and implementation of technology, including care organizations.

It becomes clear that identifying the value proposition and preconditions for implementation of UM technology in home-based dementia care together with actual key stakeholders is essential to ensure uptake and viability. To our knowledge, this study is the first to apply the business modeling perspective when doing so.

### Aim of the study

The aim of this study was to explore the values of multiple key stakeholder groups regarding UM technology to support home-based dementia care from a business modeling perspective. More specifically, the aims were:

1. To identify key stakeholders for UM technology in home-based dementia care
2. To explore and compare the value proposition of UM technology in home-based dementia care according to key stakeholders
3. To explore preconditions for successful implementation of novel UM technology in home-based dementia care

## Method

### Overview of the research approach

**Fig. 1:**
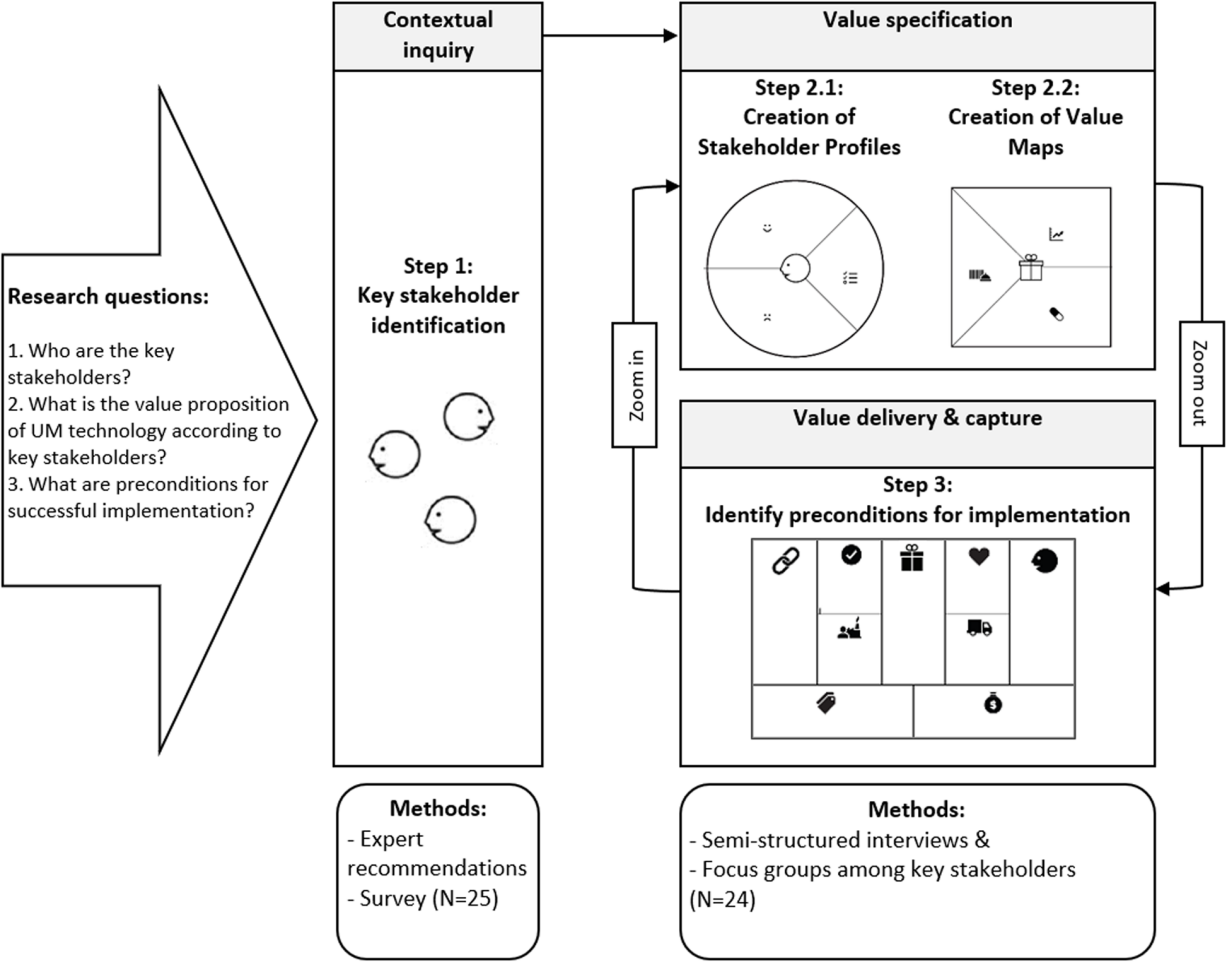
Process map of the research approach. Note: Permission was granted by Strategyzer to use the value proposition canvas and business model canvas [36] in the figure

An overview of the steps taken in this study is shown in Fig. 1. We applied a stakeholder-centered approach building upon the approaches of Lentferink et al. [33], van Limburg et al. [34], and the CeHRes Roadmap – a holistic framework which guides the development and implementation of health technologies [37].

As a first step, we identified key stakeholders for UM technology in home-based dementia care using expert recommendations and a survey. The selected key stakeholders were then involved in the second step of the study. Within that step, we applied the value proposition canvas of Osterwalder et al. [36] to zoom in on the core element of the business model canvas (BMC) [36]: the stakeholder profiles (step 2.1) which aimed at deepening our understanding of the key stakeholder groups, and value maps (step 2.2) which show how their values are translated into design-oriented needs [33, 36]. According to Osterwalder et al., products that achieve a fit between stakeholder profile(s) and value map(s) have an improved chance of uptake [36].

As a last step (step 3), based on the input from key stakeholders, we zoomed out to the context and identified preconditions for successful implementation of UM technology for home-based dementia care using the remaining components of the BMC.

### Key stakeholder identification

A stakeholder was defined as any group or organization that could affect or is affected by UM technology [37, 38]. An initial list of stakeholders was created based on the Dutch ‘’Care Standard Dementia 2020’’ [39] – a report commissioned by the Dutch Ministry of Health in collaboration with more than 20 client- and informal care organizations, professional associations, care providers, dementia networks, and insurers. Recommendations from experts in the field of Geriatrics were used to refine this list. Afterwards, to identify key stakeholders, an online survey (see Additional file 1) was distributed among potential stakeholders on the list via the research team’s professional network and spread further via snowball sampling. The survey asked participants to add stakeholders who were missing. Furthermore, participants were asked to rate each stakeholder’s relevance based on the stakeholder salience approach by Mitchell et al. [40]. Each stakeholder on the list was rated on 3 attributes on a scale from 0 (not applicable at all) to 3 (very much applicable):

1. ‘’Power’’: the amount of influence a stakeholder’s opinion will have
2. ‘’Legitimacy’’: the extent to which a stakeholder must be involved
3. ‘’Urgency’’: the extent to which a stakeholder’s needs and wishes require immediate attention

### Participant inclusion

Once the key stakeholders were identified, a combination of focus groups and semi-structured interviews (*N* = 24) were conducted to explore the value proposition and preconditions for implementation of UM technology in home-based dementia care. Recruitment of key stakeholders took place from a variety of Dutch aged care institutions and the research team’s professional network. All participants provided informed consent prior to the study.

### Focus group- and interview guide

At the beginning of each session, participants were given a demonstration of the general concept and idea of UM technology using illustrations and a standardized explanation (see Additional file 2). Subsequently, a topic guide (see Additional file 2) was used containing open-ended questions based on the value proposition canvas [36], business model canvas [36], and previous research [31]. The *stakeholder profile* contained 3 elements aimed at gaining a deeper understanding of the stakeholder: *stakeholder goals*, *gains*, and *pains.* The stakeholder goals reflected the overall goals that stakeholders aspire to achieve in the care for community-dwelling PwD or the needs they are trying to satisfy. Gains were explored by asking the participants what the added value of UM technology should be in achieving certain goals, while pains centered around the potential negative aspects that need to be avoided or form a barrier. Subsequently, we used the *value map* to gain insight into how stakeholders believed their gains and pains should be translated into design-oriented needs. We asked questions related to *gain creators* (e.g. What is needed to realize a certain gain?) and *pain relievers* (e.g. What is needed to relieve a certain pain?). The last part of the sessions focused on preconditions for successful implementation of UM technology in home-based dementia care for which the remaining components of the business model canvas were used as a guide (key partners, key activities, key resources, end-user relationships, end-user segments, channels, costs, revenues). Like van Limburg et al. [34], we zoomed out to the context within which the technology will be implemented and asked for success factors, potential barriers, and specific prerequisites. For all stakeholder groups, questions and procedures were globally the same, except that the questions were tailored to their background. The value proposition part was addressed in all stakeholder sessions, whereas the part on implementation was addressed in those sessions including stakeholders with a (care-)managing, policy-advising, or formal caregiving role. All sessions were audio-recorded and lasted about 1.5 h each.

### Data analysis

To determine the rank of the different stakeholders, the total score based on the 3 attributes power, legitimacy, and urgency was calculated.

The audio recordings of the focus groups and interviews were transcribed verbatim and qualitative content analysis was performed using the software package Atlas.ti 9. The value proposition of UM technology for home-based dementia care was analyzed separately for each stakeholder group. First, relevant quotes were selected and categorized into one of the main categories: (1) stakeholder goals, (2) gains, (3) pains, (4) gain creators, and (5) pain relievers. Subsequently, selected quotes were further categorized inductively into overarching codes. To analyze preconditions for successful implementation, relevant quotes from focus groups with (care) managers, policy advisors, and care professionals were categorized into one of the components of the BMC (key partners, key activities, key resources, end-user relationships, end-user segments, channels, costs, revenues). Thereafter, selected quotes were further categorized inductively into codes. To minimize single-researcher bias, a second researcher (AB) independently coded 10% of the data and tested whether the codes described were proper interpretations of the data. Points of disagreement were discussed until consensus was reached between the two researchers (CW and AB) which resulted in the final coding scheme.

## Results

### Key stakeholders for UM technology in home-based dementia care

In Table 1, the results of the key stakeholder identification survey (*N* = 25) are shown. The total mean relevance score for stakeholders was 1.7 (SD = 0.3) with a range of 1.1 to 2.3. Stakeholders with a relevance score >  = 2.0 were defined as key stakeholders as they represented the top third of all 27 stakeholders (ranks 1–3). The identified key stakeholders generally represented potential end-users (informal caregivers, home care professionals, and PwD) as well as managers and policy advisors who typically have a formal role in decisions for or against the purchase (or reimbursement) of care technology. Notably, the survey results revealed that medical care specialists such as general practitioners or clinical geriatricians were generally not seen as key stakeholders.

**Table 1:** Stakeholder ranking according to the stakeholder identification survey

Stakeholder^b^	Relevance stakeholder (Mean)				Rank^a^
	**Power (0–3)**	**Legitimacy (0–3)**	**Urgency (0–3)**	**Total M (SD)**	
**Informal caregivers**					
Informal caregivers living together with PwD	1.7	2.6	2.6	2.3 (0.4)	1
Informal caregivers caring at a distance for PwD	1.6	2.4	2.4	2.1 (0.4)	2
**Care recipients**					
Community-dwelling people with dementia or mild cognitive impairment (MCI)	1.2	2.5	2.6	2.1 (0.6)	2
**Health insurance companies**					
Policy advisors aged care	2.5	2.0	1.8	2.1 (0.3)	2
**Aged care institutions**					
Policy advisors care technology & innovation	2.1	2.4	1.8	2.1 (0.2)	2
Director/ general manager	2.4	2.0	1.5	2.0 (0.4)	3
Manager home care	2.0	2.1	1.8	2.0 (0.1)	3
IT administrator	1.8	1.8	1.8	1.8 (0.0)	4
Chief operating officer	1.9	1.6	1.2	1.6 (0.3)	6
**Home care professionals**					
Case manager dementia	1.7	2.3	2.3	2.1 (0.3)	2
District nurse	1.5	2.2	2.1	2.0 (0.3)	3
Personal care assistant	1.1	1.9	1.8	1.6 (0.4)	6
Paramedics (e.g. physiotherapist, occupational therapist)	1.1	1.5	1.3	1.3 (0.2)	9
**Science**					
Technical engineers	1.6	1.9	1.7	1.7 (0.1)	5
Ethical scientists	1.1	1.8	1.5	1.5 (0.3)	7
**Municipalities**					
Assessor care indications	1.5	1.8	1.4	1.6 (0.2)	6
**Business**					
Software companies	1.4	1.8	1.5	1.6 (0.2)	6
**Interest organizations**					
Patient organizations (e.g. national Alzheimer Association)	1.8	1.8	1.5	1.7 (0.1)	5
Informal care associations	1.4	1.6	1.4	1.5 (0.1)	7
Professional associations (e.g. national District Nurse Union)	1.6	1.5	1.2	1.4 (0.2)	8
**Medical care specialists**					
Aged care physician	1.6	1.6	1.5	1.6 (0.0)	6
General practitioner	1.6	1.6	1.4	1.5 (0.1)	7
General practitioner assistant (POH)	1.2	1.5	1.3	1.3 (0.1)	9
Clinical geriatrician	1.3	1.2	1.2	1.2 (0.0)	10
Neurologist	1.2	1.2	1.1	1.2 (0.0)	10
**Social work organizations**					
Social worker	1.1	1.6	1.3	1.3 (0.2)	9
Informal care support consultant	1.0	1.4	1.0	1.1 (0.2)	11

^**a**^Ranks range between 1 and 11 based on the total stakeholder relevance scores

^**b**^Additional explanations: Case manager dementia = District nurse who coordinates the care for PwD; Personal care assistant = Professional trained to assist in daily self-care (e.g. washing, eating, dressing); Aged care physician = Geriatric specialist within the nursing home; Clinical geriatrician = Geriatric specialist within the hospital; General practitioner assistant (POH) = nurse specialist in primary care

Table 2 shows an overview of included key stakeholders (ranks 1–3) for the qualitative part of this study. Similar key stakeholders were grouped together, resulting into 5 different key stakeholder groups. In general, 4–6 participants per stakeholder group were included.

**Table 2:** Overview of included key stakeholders (*N* = 24)

**Stakeholder group**	**Included stakeholders**	**Age range** **(years)**	**n**
Informal caregivers	• Informal caregivers living together with PwD (e.g. partners) • Informal caregivers caring at a distance for PwD (e.g. adult children)	42–77	5
Home care professionals	• Case managers dementia (district nurses who coordinate the care for PwD) • District nurses	38–55	5
People with dementia	• Community-dwelling people with dementia or mild cognitive impairment (MCI)	76–82	4
Directors and managers	• Directors/ general managers and program managers within aged care institutions • Managers home care	42–50	4
Policy advisors	• Policy advisors care technology & innovation within aged care institutions • Policy advisors aged care within health insurance companies and at governmental level (Dutch Ministry of Health)	27–46	6

### The value proposition of UM technology for home-based dementia care

**Fig. 2:**
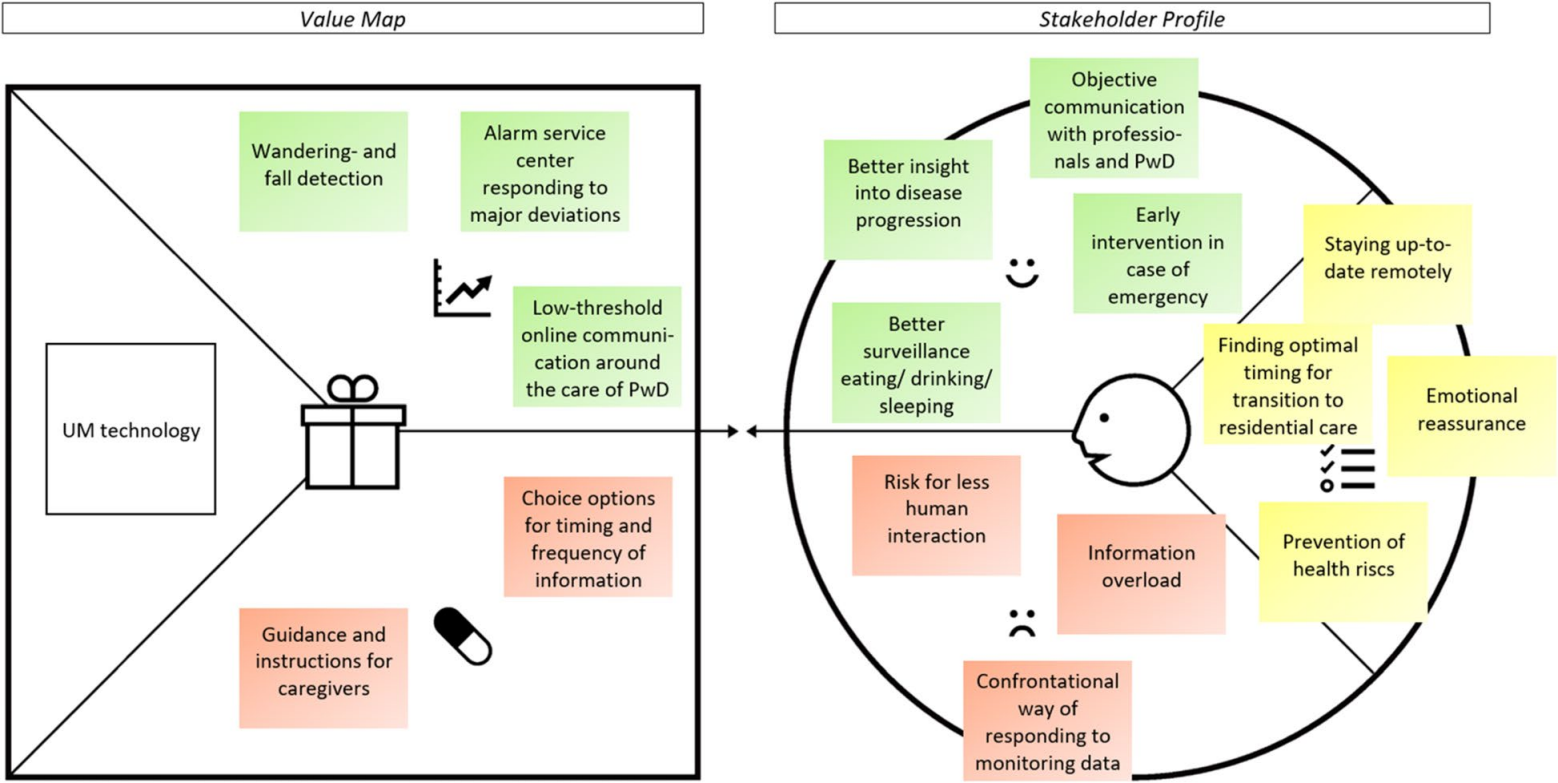
The value proposition of UM technology according to informal caregivers of PwD. Note: The stakeholder profile (right side) contains stakeholder goals (yellow), gains (green), and pains (red). The value map (left side) shows what is needed from UM technology to realize the gains and relieve the pains. Permission was granted by Strategyzer to use the value proposition canvas [36] in the figure

**Fig. 3:**
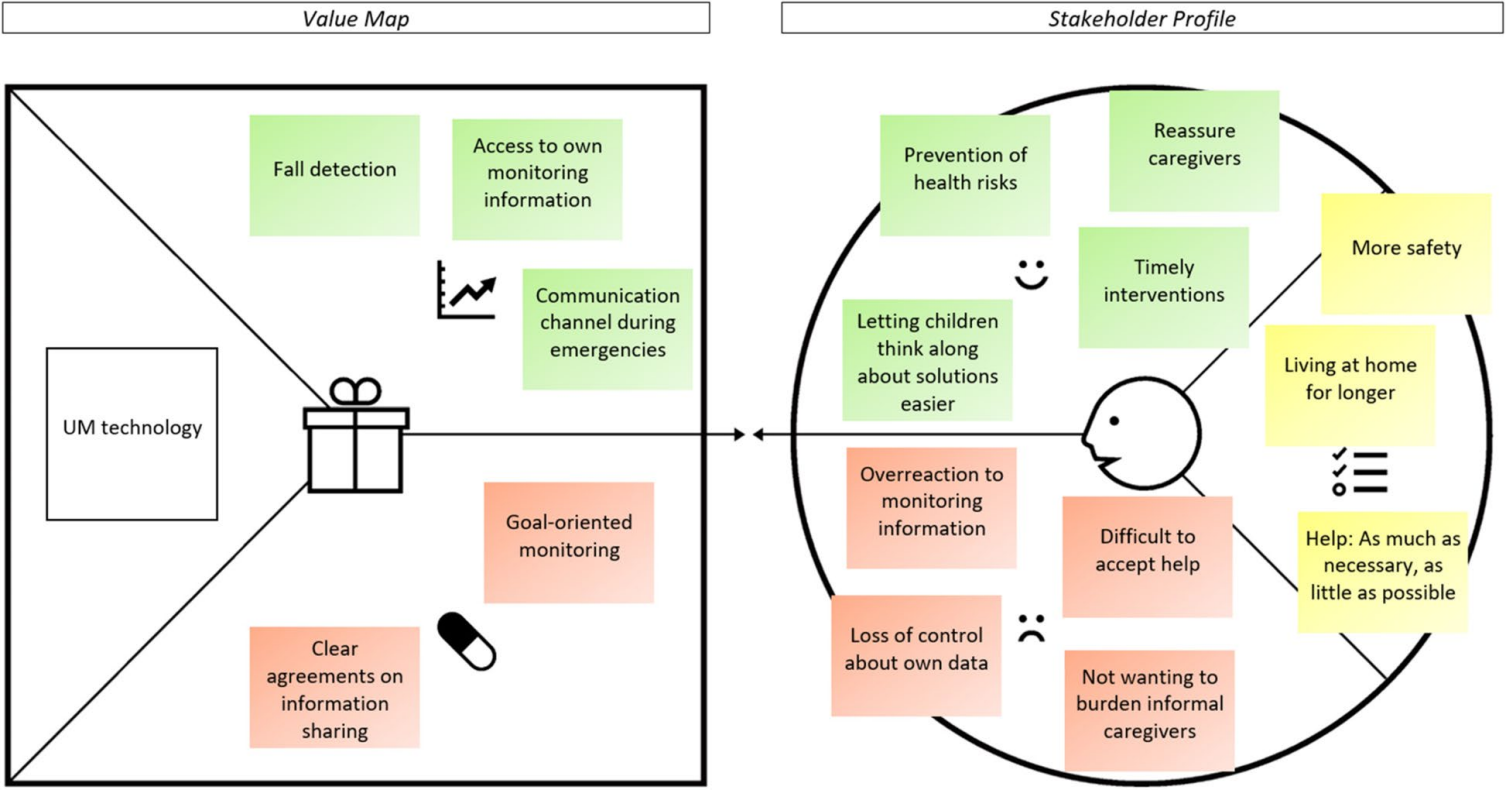
The value proposition of UM technology according to PwD. Note: The stakeholder profile (right side) contains stakeholder goals (yellow), gains (green), and pains (red). The value map (left side) shows what is needed from UM technology to realize the gains and relieve the pains. Permission was granted by Strategyzer to use the value proposition canvas [36] in the figure

**Fig. 4:**
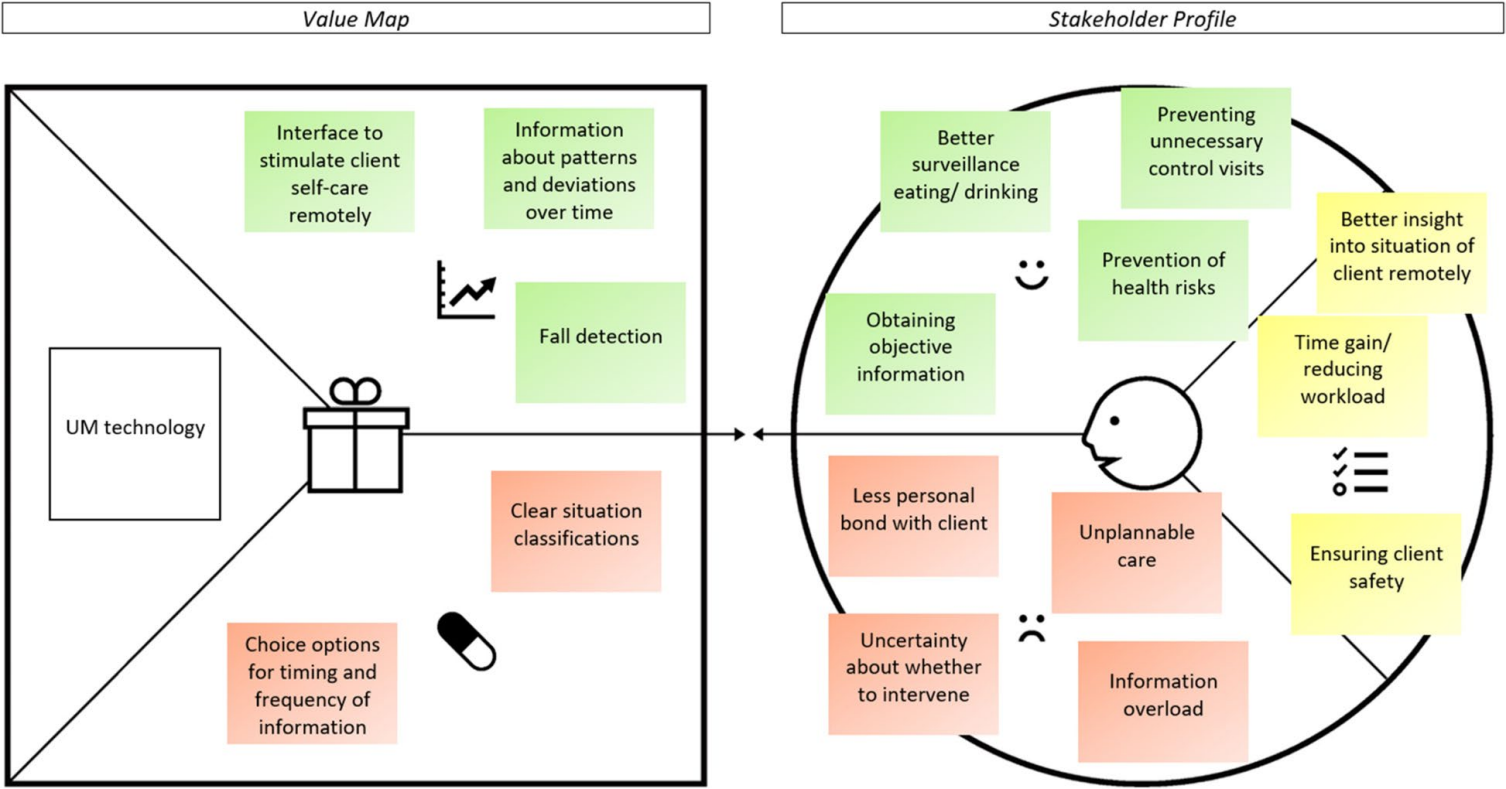
The value proposition of UM technology according to home care professionals of PwD. Note: The stakeholder profile (right side) contains stakeholder goals (yellow), gains (green), and pains (red). The value map (left side) shows what is needed from UM technology to realize the gains and relieve the pains. Permission was granted by Strategyzer to use the value proposition canvas [36] in the figure

**Fig. 5:**
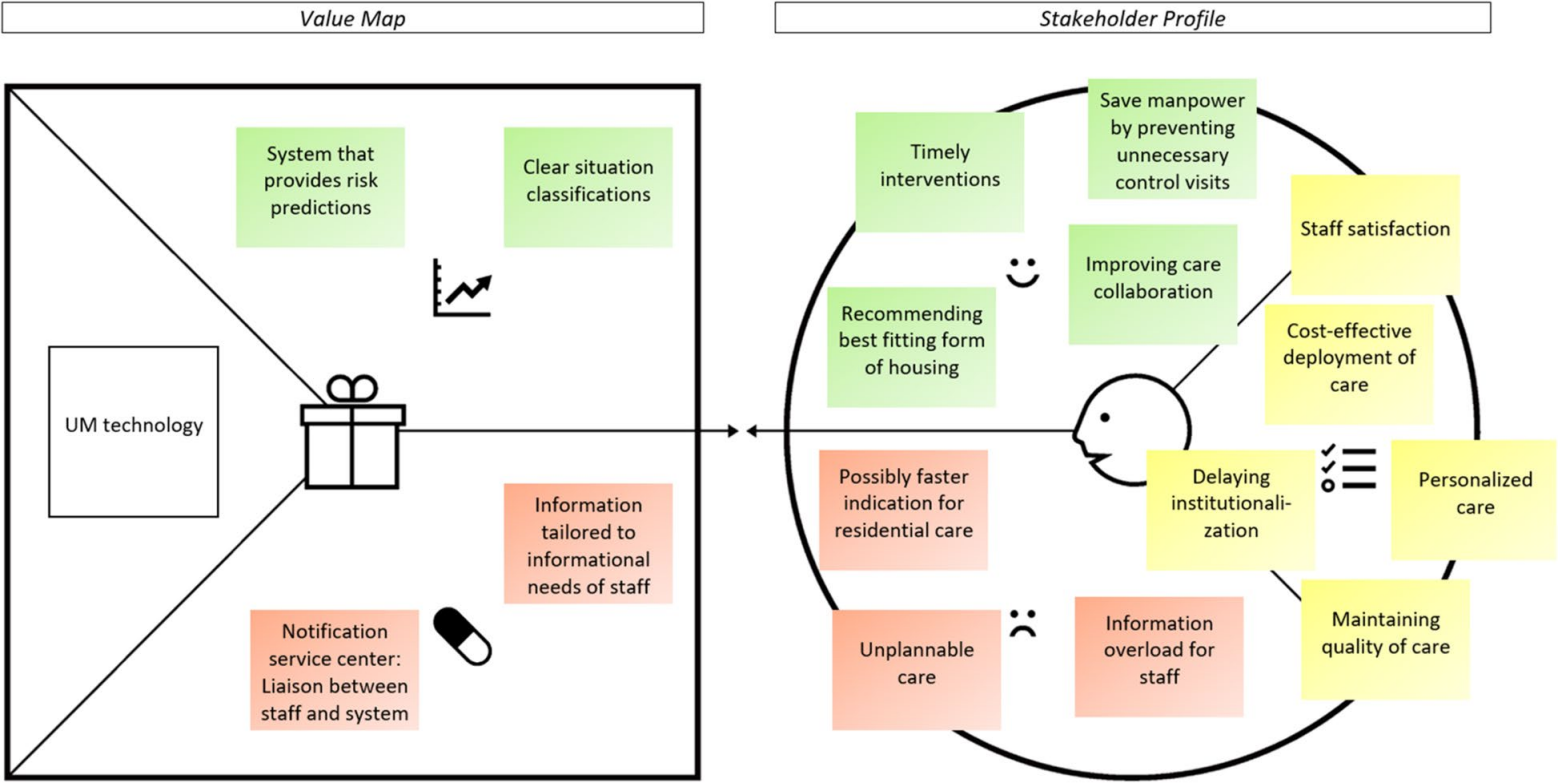
The value proposition of UM technology according to directors, managers, and policy advisors. Note: The stakeholder profile (right side) contains stakeholder goals (yellow), gains (green), and pains (red). The value map (left side) shows what is needed from UM technology to realize the gains and relieve the pains. Permission was granted by Strategyzer to use the value proposition canvas [36] in the figure

In the following part, the value proposition (stakeholder profile and value map) of UM technology is presented per key stakeholder group. As can be seen in Figs. 2, 3, 4 and 5, the stakeholder profiles contain the most important *stakeholder goals* (yellow), *gains* (green), and *pains* (red). The value maps show what, according to stakeholders, is needed from UM technology to realize the most important gains (*gain creators*: green) and relieve the most important pains (*pain relievers*: red). The value propositions for directors/ managers and policy advisors were merged as those groups showed very similar results.

#### Informal caregivers of PwD

##### Stakeholder profile

As can be seen in Fig. 2, informal caregivers of PwD would like to be able to *stay up-to-date remotely* about the situation of their loved one. UM technology should contribute to gaining a *better insight into the disease progression*, but also goes along with a *risk for less human interaction*.

*‘’I think the danger of less human contact is very important [...] As soon as things go well according to the system, someone might come by less often when it is busy.*’’ (caregiving daughter, age 55)

Next to that, the *prevention of health risks* is a priority for informal caregivers. According to them, this can mainly be achieved by *better surveillance of eating, drinking, and sleeping:*

*''That [the system] provides insight and structure into what someone still eats and possibly drinks.''* (caregiving daughter, age 52)

*''It is reassuring if you can check via this system how it goes, especially at night.''* (caregiving daughter, age 55)

On the other side, according to informal caregivers, the use of UM technology should not result in *information overload* and a *confrontational way of responding to monitoring data.*

*‘’It can also become a kind of big brother and that people with dementia are no longer allowed to make their own choice. The caregiver should not immediately correct [behavior], that is a danger.’’* (caregiving daughter, age 52)

Besides this, informal caregivers expressed a great need for *emotional reassurance* on times they cannot be together with their loved one and hoped that UM technology can contribute to this by enabling *early intervention in case of emergency*.

Lastly, informal caregivers often find it difficult to determine the *optimal timing for transition to residential care* together with their loved one and professionals. They expect UM technology to provide information that enables more *objective communication with professionals and their loved one*:

*‘’When you have data, you can look at situations objectively without emotion […] And then I think it is easier to say we have done everything we can, but now living at home is not responsible anymore.’’*(caregiving daughter, age 43)

##### Value map

To realize the gains and relieve the pains of UM technology, informal caregivers emphasize several aspects. To be able to intervene early in case of emergency, *wandering- and fall detection* is an essential feature. According to them, such information can best be delivered via an *alarm service center responding to major deviations* in the monitoring data by calling down a pre-defined set of responsible contact persons.

*‘’If there is an emergency, I would like to be called immediately. If that is not possible, the second contact person must be called.’’* (caregiving daughter, age 52)

To realize objective communication with care professionals and loved ones, informal caregivers would desire a *low-threshold online communication* medium centered around the care of their loved one and connected to the UM technology.

*''I think that a platform works well, you can read in the record what is happening, and you can also ask questions to which you will receive an answer in the short term.''* (caregiving daughter, age 52)

Information overload can, according to informal caregivers, mainly be relieved by *choice options for timing and frequency of information*:

*''I would like to decide for myself when I want to receive the information. It may also change, depending on the situation.''* (caregiving daughter, age 52)

Lastly, to prevent using UM technology in a confrontational or otherwise undesired way, informal caregivers would appreciate *guidance and instructions* about respectful ways of interaction with their loved one before using the technology.

#### People with dementia

##### Stakeholder profile

As can be seen in Fig. 3, people with dementia expressed their wish for *more safety* at home. According to them, UM technology should help to *prevent health risks*.

*''When I am alone, it's nice to know that there is no situation in which I will fall and that nobody knows that.''* (female, age 76)

At the same time, according to PwD, an *overreaction to monitoring information* should be avoided, i.e. a situation in which caregivers start to interfere too much and thus disrupt PwD’s lives.

PwD generally wished to be able to *live at home for longer*, especially through *timely interventions*. However, they often find it *difficult to accept (extra) help* because they already give away a lot of (physical) privacy:

''P: You have to give away more and more, I don't like it at all.

*R: Give away?*

P: Yes, getting things done by others more and more, letting them help you, I don't really want that.'' (male, age 78)

PwD would therefore like to receive *as much help as necessary, but as little as possible*. In that way, they hope that the use of UM technology will make it easier to *reassure caregivers*:

*“It [the monitoring information] can be useful for the person to whom it is told to know whether things are going well.”* (male, age 82)

By using UM technology, PwD also hope to *let their children think along about solutions easier* and to involve them more closely in certain care choices. Two pains are connected to this: First, PwD do *not want to burden their informal caregivers*. Adult children for instance often have their own families and PwD do not want them to feel like they always have to respond to monitoring information. Second, PwD see a danger of *losing control about their own data* which could be shared with others without permission.

##### Value map

To realize the gains of UM technology, PwD emphasize a number of aspects. First, *fall detection* is seen as a useful feature, which is also acceptable in the bathroom to detect slips:

*''Well, if my wife has a device that could record these kinds of things, then I don't have a problem with that. Slipping [in the bathroom] is an accident. The system then wants to help me, so it's okay.''* (male, age 82)

For emergency situations, a *communication channel* between the PwD and (in)formal caregiver is seen as helpful:

*''If I have fallen and people can communicate with me, I would like that. But only in that case.''* (female, age 76)

Besides this, the own monitoring information should generally be *accessible* to PwD.

To relieve the pains of UM technology, PwD emphasize two aspects: First, the use of the technology should be *goal-oriented*: PwD find it important that the usefulness of measurements is clear to them. Second, according to PwD, there should be *clear agreements on with whom the monitoring information is shared.*

#### Home care professionals of PwD

##### Stakeholder profile

As can be seen in Fig. 4, home care professionals of PwD would like to achieve *better insight into the situation of clients with dementia remotely*. UM technology should enable *better surveillance of eating/ drinking*:

''P1: Eating and drinking is of course stimulated, but it is about those moments when the nurses are not there, and the day is quite long.

*P3: Then you're actually talking about a surveillance problem [...] And sitting next to someone for half an hour to see if they are eating is not possible.''* (P1: case manager dementia; P3: district nurse)

However, a *less personal bond with clients* on the other hand should not be the result of UM technology. In addition*, time gain/ reducing workload* is an important goal of the home care professional. They hope to be able to *prevent unnecessary control visits* to clients with dementia by using UM technology. According to them, too much travel time is currently wasted controlling self-care and sleep on moments where this would have been unnecessary:

''P3: You're not going to wake someone up to see if they're still in bed, that's absurd […]

P2: And otherwise you drive there for only 10 min.

P3: [...] And the routes in the evening, that are 35 people that you need to visit. Getting in and out of the car 35 times, that's a workout in itself.'' (P3: district nurse; P2: case manager dementia)

On the other hand, home care professionals also see an increased risk of *unplannable care* due to UM technology which disseminates real-time monitoring data. An abrupt increase of just-in-time care moments in response to monitoring data opens up a dilemma: The professional would like to provide the extra care but can face a lack of sufficient resources (manpower) to be able to do this.

''P1": As a home care team, you normally have your care moments on which you deliver care. But unplanned care moments […] if those suddenly increase…

*P2: Yes that is a disadvantage if you use this system. When do you visit the client? There must be staff who is able to respond.''* (P1 & P2: case managers dementia)

Furthermore, home care professionals see *ensuring client safety* as a central task. They see UM technology as a tool for *obtaining objective information* to support the *prevention of health risks* of PwD.

''P3: Not eating and drinking too little, those are all safety aspects that you can't make objective currently. It's all feelings […]

*P1: You do have a gut feeling, but if you receive such [monitoring] information, then that is correct.* (P3: district nurse; P1: case manager dementia)

Two pains are connected to this: *uncertainty about whether to intervene* based on UM technology notifications, and the danger of *information overload*.

##### Value map

To realize the gains of UM technology, home care professionals emphasize several aspects. To reduce unnecessary control moments they expect UM technology to not only monitor but also provide an *interface to stimulate client self-care remotely*:

''I am not only thinking of supervision but also stimulation, you know, with speech […] Just a big screen showing the district nurse saying, 'Will you grab your drink from the fridge?' […] I think you should stimulate more remotely instead of going to the client.'' (district nurse).

In addition, *fall detection* and *information about patterns and deviations over time* are seen as essential features by home care professionals.

''R: And we also talked about the day-and-night rhythm.

*P3: Yes if that changes you should be able to compare periods. Then it can certainly be useful.''* (district nurse)

To relieve the pains of UM technology, home care professionals expect such systems to provide *clear situation classifications* making it easy to determine whether to intervene based on the monitoring data. Lastly, to prevent information overload, UM technology should provide choice options for *timing and frequency of information*.

#### Directors, managers, and policy advisors

##### Stakeholder profile

As can be seen in Fig. 5, the group of directors, managers, and policy advisors considers the *cost-effective deployment of care* and *staff satisfaction* as essential goals. The use of UM technology should *save manpower by preventing unnecessary control visits* to clients with dementia.

*''And based on that I wonder: does the system save manpower and time? Especially when we talk about the unnecessary contact moments''* (policy advisor Dutch Ministry of Health)

Just like home care professionals, this group also sees an increased risk of *unplannable care* due to UM technology which disseminates real-time monitoring data. This can be problematic, especially during peak times when capacity of staff is limited.

Also, *personalized care* is among the goals of directors, managers, and policy advisors. According to them, UM technology should help in *recommending the best fitting form of housing* to clients with dementia.

Next to that, *maintaining quality of care*, and *delaying institutionalization* of PwD (in connection to a limited residential care capacity) are essential goals for this group. UM technology should contribute to this by enabling *timely interventions*:

''P4: If the system detects issues sooner, you are on time with interventions.

*P3: Yes exactly, you can discover gaps that you can then fill again so that staying at home becomes feasible and safe again.''* (P4: policy advisor Dutch Ministry of Health; P3: manager home care)

However, according to directors, managers and policy advisors, there is a field of tension: UM technology should help PwD to live at home for longer, but it can just as well work the other way around: Through better surveillance, a *faster indication for residential care* may be possible.

Lastly, participants highlighted the potential of UM technology to *improve care collaboration* among professional caregivers, however, also acknowledged the risk of *information overload for staff*:

*''Information overload might kill the chance of success of this system.''* (program manager)

##### Value map

To realize the gains and relieve the pains of UM technology, directors, managers, and policy advisors emphasize several aspects. To anticipate a client’s future situation and initiate timely interventions, they expect UM technology to provide intelligent *risk predictions*. In that way, the technology might compensate for a lower level of expertise of care staff, which becomes relevant in the light of an increasing shortage of highly qualified staff.

''P3: You can also detect risks in advance, including falls and things like this. From the data, you might predict and anticipate this […]

*P1: And that has a twofold consequence: On the one hand you can reduce the risk of clients becoming ill. But at the same time - when there is shortage on the labor market - the system can support staff with a low level of expertise in providing optimal care.''* (P3: policy advisor health insurance company; P1: director aged care institution)

Also, UM technology should provide *clear situation classifications*, making it easy for care staff to distinguish between acute and non-acute situations. To prevent information overload, participants favored the idea of a *notification service center* which acts as a liaison between care staff and the monitoring system and informs staff in case any actions need to be taken:

''P4: Perhaps you can set up a service center that responds to the monitoring information and informs the professional if something is wrong.

*P3: On moments when something is needed.''* (P4: policy advisor Dutch Ministry of Health; P3: manager home care)

Lastly, participants stressed the importance of *monitoring information tailored to the informational needs of staff*.

### Convergent and divergent perspectives between stakeholders

Stakeholders generally agreed that UM technology should provide gains in terms of obtaining more objective information, better surveillance of eating/ drinking/ sleeping of PwD, timely interventions, and prevention of health risks and unnecessary control visits, whereas often mentioned pains included less human interaction, information overload, and unplannable care due to real-time monitoring. A field of tension emerged mainly between the care-providing/ care-managing stakeholders and the group of PwD: While timely interventions and the prevention of health risks were shared expected gains of UM technology, we found that it is often difficult for PwD to accept this (extra) help and the infringement on privacy associated with it. PwD indicated to be afraid of a disruption of daily life due to over-involvement of caregivers.

In terms of design-oriented needs (value maps), stakeholder groups most often highlighted the need for fall- and wandering detection, an alarm/ notification service center responding to major deviations in the monitoring data, choice options for timing and frequency of information, and clear situation classifications making it easy for caregivers to distinguish between acute and non-acute situations. The group of directors, managers, and policy advisors was the only one that expected UM technology to also provide risk predictions to better anticipate a client with dementia’s (future) situation.

### Preconditions for successful implementation

**Fig. 6:**
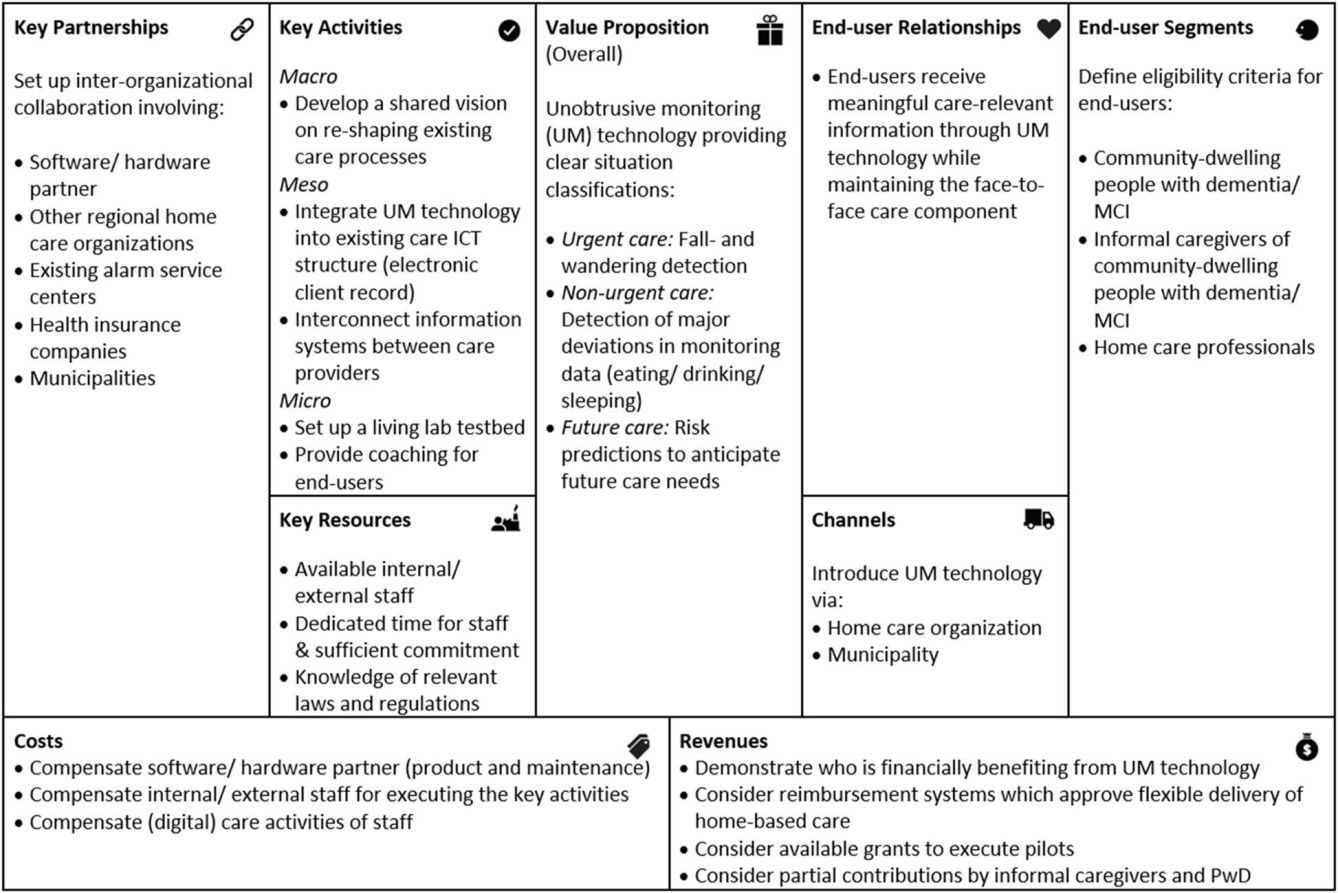
Overview of preconditions for successful implementation of UM technology in home-based dementia care using the business model canvas. Note: Permission was granted by Strategyzer to use the business model canvas [36] in the figure

Figure 6 shows an overview of what is needed to realize successful implementation of UM technology in home-based dementia care according to consulted stakeholders (directors, managers, policy advisors, and home care professionals). Findings are structured according to the business model canvas, taking the perspective of the implementing home care organization that wants to offer UM technology to their clients with dementia. The 9 elements of the model summarize preconditions related to the needed infrastructure (top left 3 elements), end-users (top right 3 elements), and costs and revenues (bottom 2 elements), required to deliver the (overall) value proposition (middle element).

#### Overall value proposition

Based on our multiple stakeholder value maps that have been described earlier, the overall value proposition could globally be summarized as follows: In essence, UM technology for home-based dementia care should generally provide clear situation classifications via a notification center/ platform that helps caregivers to adequately respond to the PwD’s care needs. Situations include a) *Urgent care*: fall- and wandering detection requiring immediate intervention, b) *Non-urgent care*: detection of major deviations of monitoring data (eating/ drinking/ sleeping) requiring timely intervention, and c) *Future care*: Risk predictions to anticipate future care needs.

#### End-user segments

According to stakeholders, successful implementation of UM technology starts with *defining clear eligibility criteria for end-users*, including community-dwelling PwD, their informal caregivers, and home care professionals. Related to the eligibility of PwD, the right moment to introduce UM technology in the disease pathway was perceived before an official diagnosis is present. Consequently, stakeholders suggested the technology should be offered as both pre- and post-diagnostic support, with potential users ranging from persons with mild cognitive complaints to those already on the waiting list for admission into residential care.

*‘’R: But the question is also: when do you actually start monitoring in the home situation?*

*P3: Very early, and actually much earlier than the onset of the disease, I think. So I think that should already become part of life for vulnerable older adults.''* (P3: district nurse)

#### Key partnerships

Stakeholders’ overarching message was not to implement a stand-alone service together with the *software/ hardware partner* but to *set up a broader inter-organizational collaboration* to successfully implement UM technology in home-based dementia care. Consequently, stakeholders considered different partners to be helpful. First, they highlighted the need for collaboration between *regional home care organizations*. The implementation of UM technology as a tool to facilitate personalized care may lead to greater fluctuations in care provision. In connection to the increasing shortage of staff, it is therefore preferable to join forces of several home care organizations to form a supporting network for exchanging manpower.

*''You have to arrange certain things together, especially because there will be less staff in the future. And then you could merge certain services together, as one organization [...] This can be done with many organizations so that people can live at home for longer.''* (policy advisor health insurance company)

*''That's what I mean by collaboration, which is very important. We often talk about self-management, but I also think that ‘together-management’ is important to enable living at home for longer and that such a [monitoring] system can be supportive in this.''* (policy advisor care technology & innovation)

In addition, stakeholders considered it essential to partner with already existing and widely accepted *alarm service centers* which respond to clients with an emergency button at home.

*''I think the system should be implemented with several collaborations, for instance, the alarm service center.’’* (case manager dementia)

*''There is already a lot of structure, such as a contact center that can play a role.''* (manager home care)

Lastly, stakeholders recommended the involvement of *health insurance companies* and local *municipalities* as important partners, as they considered the implementation of technology to support extended independent living of PwD as a joint societal and regional effort.

#### Key activities- and resources

Stakeholders mentioned several activities to be carried out to achieve successful implementation of UM technology in home-based dementia care. On the macro-level, they considered the *development of a shared vision on re-shaping existing care processes* as important. Stakeholders valued the idea to deliver personalized, flexible care based on UM technology which disseminates real-time information and provides risk predictions. However, this also requires a shift in how home care professionals work: From a scheduled to more flexible care provision practice.

On the meso-level, the *integration of UM technology into the existing care ICT structure* was seen as essential. Home care professionals would like to minimize the number of systems they work with. A platform providing access to monitoring information must therefore be integrated into existing electronic client records. At the same time, stakeholders wished for *interconnected information systems between care providers* to facilitate data-driven care collaboration.

*''Integrated would be the best. We would like to work with as few different systems as possible and if those can be connected, that is always a good thing to do.''* (manager home care)

*''You have to connect with the ICT of other organizations and your own electronic client record.''* (program manager)

One of the overarching messages of stakeholders was to ‘’think big and start small’’. On the micro-level, stakeholders therefore suggested to *set up a living lab testbed* which enables to experiment with UM technology on a small scale.

*''I think you need living labs, trying things out [...] To also have good conversations with staff about what works and what doesn't work.''* (director aged care institution)

Lastly, *providing coaching to end-users* (staff and clients) was seen as essential. Care staff, especially, must be sufficiently prepared, trained, and stimulated to work in a different way by using UM technology.

*''What is needed is also very good coaching. In the beginning especially for the professional, the informal caregiver, and perhaps the client too.''* (policy advisor Dutch Ministry of Health)

*''The role of the district nurse is going to change; they will analyze data and adjust plans accordingly.''* (manager home care)

Key resources needed to conduct the key activities include *available internal/ external staff* with *dedicated time and sufficient commitment*, and *knowledge of relevant laws and regulations* (such as, e.g., the European General Data Protection Regulation).

#### Channels and end-user relationships

According to stakeholders, UM technology can be introduced via different channels. According to them, the most ideal way is via the *home care organization*, letting the technology become part of the regular portfolio of care options. In that way, however, community-dwelling PwD who do not (yet) receive professional home care are out of reach. Stakeholders therefore suggested collaborating with *municipalities* to reach those who do not yet receive professional home care or are not yet officially diagnosed with dementia. As stated by stakeholders, the introduction of UM technology via the private sector as an ‘’off the shelf’’ device is not an ideal option. For most PwD, professional home care usually becomes needed at a given moment. It can then be too demanding for care professionals to have to adjust to various systems purchased by clients and informal caregivers.

*''What is important is that offering monitoring technology should be linked to one of the care organizations. The professionals of the home care organization then simply have it in their package, so it becomes part of the regular offer [...] It could also be that a highly educated, wealthy informal caregiver just buys it and says ''Could you please work with it?'' That doesn't go well in my opinion [...] Then you may miss the connection with how a professional performs his role.''* (policy advisor Dutch Ministry of Health)

Ideal end-user relationships were generally described as *receiving meaningful care-relevant information through UM technology while maintaining the face-to-face care component*.

#### Costs and revenues

To achieve sustainable implementation of UM technology, the overarching message was to *demonstrate who is financially benefiting from the technology* and involve those parties in covering the main cost drivers (*compensations for software/ hardware partner,* for *internal/ external staff executing the key activities,* and for *(digital) care activities of staff*).

*''You have to get the business case right and see who picks the fruits and let them contribute to the financing.''* (policy advisor care technology & innovation)

What makes this challenging is that it is not always clear which care sector will financially benefit from UM technology. Whereas the technology might reduce costs in home-based care due to a possible time gain, it might also reduce costs in hospital care by preventing adverse health outcomes. Nevertheless, according to stakeholders, the reimbursement of technology-supported home-based care is often easier to accomplish, when costs and benefits stay within the same care sector.

In the Netherlands, multiple care reimbursement systems exist. An important message was to *consider reimbursement systems which approve flexible delivery of home-based care*. The implementation of UM technology as a tool to facilitate flexible, just-in-time care delivery to PwD also asks for flexible reimbursement of care. Home care professionals would like to be able to react to UM technology notifications and visit clients with dementia when it is most needed. This flexible deployment of home care (meaning the up- or downscaling of care in the short term) is, however, not possible within all Dutch reimbursement systems.

*''R: And what happens if something changes in the care situation in the short term? For example, if we go back to a real-time monitoring system and it turns out that care must be added because you see it in the system, how exactly does that work?*

*P: Within the health insurance it actually goes very quickly, the district nurse indicates more [care] which can be delivered the next day immediately [...] Within the WMO [municipality support] or the WLZ [long term care] it is a problem, because then you have to apply for it, then people need to look at it, and then it's a few weeks later.''* (P: policy advisor care technology & innovation)

Lastly, stakeholders recommended to *consider available grants to execute pilots* with UM technology, and *partial contributions by informal caregivers and PwD* which were seen as acceptable as long as the technology provides added value to them. This might also help home care organizations to win back investments on the technology self (software/ hardware) which are normally not covered by care reimbursement systems.

*''There are always posts that clients have to pay themselves, that is not weird. If there is added value for the informal caregiver and client, then it is not weird at all to think about that.''* (policy advisor care technology & innovation)

## Discussion

### Principal findings

This study aimed to explore the values of multiple key stakeholders regarding unobtrusive monitoring (UM) technology to support home-based dementia care from a business modeling perspective. With our multimethod approach, we were able to identify and rank key stakeholders, discover their value proposition towards UM technology, and elicit preconditions for successful implementation.

Extending own previous research on the needs of (in)formal caregivers of PwD [31], our study helps to understand a broader range of relevant stakeholder perspectives towards the newest generation of UM technology. Also, our study responds to a recent literature review [41] which advised to further explore preconditions for implementation which are not only tailored to the context of home-based dementia care but also tailored to specific kinds of technology.

Our study identified key stakeholders for UM technology in home-based dementia care including potential end-users, directors and managers within home care, and policy advisors within the aged care and health insurance sector. Interestingly, medical care specialists such as general practitioners or clinical geriatricians were generally not identified as key stakeholders. However, this does not necessarily mean that such stakeholders might not become important. The trend towards care collaboration and integrated dementia care [42] suggests that those specialists might affect or be affected by UM technology in the future.

Furthermore, our results revealed stakeholder profiles which deepened our understanding of their goals, and gains and pains towards UM technology. In general, stakeholders saw the technology as a tool to facilitate personalized timely care and which may help to save caregivers’ resources. Privacy and ethical issues mentioned (such as the loss of control about own data and less human interaction) were seen as preventable risks rather than a given fact. However, it should be noted that specific ethical challenges related to AI exist which were not mentioned by our participants. For instance, developers of future AI-driven UM technology should be aware that FAT (fairness, accountability, and transparency) of algorithms is of growing importance, especially in scenarios concerning human health and wellbeing [43, 44].

We also recognized differences between stakeholder groups. Typical for the group of directors, managers, and policy advisors seemed to be their future-oriented focus of goals (e.g. cost-effective deployment of care, time gain, maintaining quality of care) as opposed to the goals of (in)formal caregivers and PwD which often focused on the short term (immediate benefits). A field of tension emerged mainly between the care-providing/ care-managing stakeholders and the group of PwD: While timely interventions and the prevention of health risks were shared expected gains of UM technology, we found that it is often difficult for PwD to accept this (extra) help and the infringement on privacy associated with it. This tension could lead to stakeholder dissonance which can threaten the technology’s viability if left undetected and unresolved [45, 46]. Godwin et al. [47] developed an ethical checklist for caregivers of PwD which may help to make such tensions discussable and form a shared decision for or against assistive technology use.

Although stakeholder groups expressed different design-oriented needs (value maps), those could globally be summarized into the need for clear situation classifications including urgent care (fall- and wandering detection), non-urgent care (detection of major deviations in eating, drinking, and sleeping of PwD), and future care (risk predictions to anticipate future care needs). Risk predictions to anticipate a PwD’s future situation, in particular, have been highlighted by our participants as a possible way to compensate for a lower level of expertise of care staff which becomes relevant in the light of the increasing shortage of highly qualified staff. This idea corresponds to recent policy advice on digitalization given to the Dutch Ministry of Health which highlights the potential of intelligent AI-based technology to support low-level care staff in providing higher-level care and expand their task repertoire [48]. However, a previous study among (in)formal caregivers of PwD [31] taught us at the same time that over-reliance on AI-driven UM technology should be prevented. For home care professionals, there can be a fine line between feeling supported and feeling determined by intelligent UM technology [31].

Our study provided an overview of preconditions for successful implementation of novel UM technology in home-based dementia care from the home care organization’s perspective. Essential preconditions for successful implementation mainly included inter-organizational collaboration, a shared vision on re-shaping existing care processes, integrated care ICT infrastructures, clear eligibility criteria and coaching for end-users, and reimbursement systems which approve flexible delivery of home-based care. Our results can be seen in the light of the NASSS framework [49], which shows that successful implementation of health technologies is influenced across multiple domains (condition, technology, value proposition, adopters, organizations, wider context, and embedding and adaptation over time). Most preconditions identified in our study relate to what the NASSS framework defines as the organizational context (e.g. extent of change needed to routines, work needed to implement change) and the wider economic context for successful rollout of health technology [49]. The NASSS framework has previously been applied in a literature review [41] to identify factors influencing the implementation of technology for home-based dementia care. While this review provided a useful overview, it also uncovered a knowledge gap regarding success factors for implementation related to the organizational and wider context [41]. Our findings reduce this knowledge gap in the context of UM technology for home-based dementia care.

All in all, the recommended preconditions brought forward by our participants highlight the complexity of implementing UM technology as a new digital infrastructure to support home-based dementia care and facilitate data-driven care collaboration. For instance, to facilitate data exchange between home care organizations, political/regulatory decisions will be needed. Furthermore, UM technology which disseminates real-time information within the care network of PwD requires new ways of working, characterized by a shift from a scheduled to more flexible care provision practice**.** This may help caregivers to respond to PwD who need support in the moment and prevent unnecessary control visits. However, one of the most dominant bottlenecks found in this study was the financing. The implementation of UM technology as a tool to facilitate more flexible care delivery to PwD can financially be challenging when reimbursement systems do not allow for flexible financing of care. This issue can likely be generalized to any form of data-driven just-in-time care and calls for more flexible financing structures in order to successfully implement this form of care in the future. Furthermore, our results imply that, without external grants, it can be financially challenging for home care organizations to start experimenting with innovations, including UM technology. However, in the Netherlands, health insurance companies are increasingly starting to invest in innovative pilot projects [50].

### Strengths and limitations

A strength of this study lies in the use of well-established frameworks to identify key stakeholder values and preconditions for successful implementation. Developing health technologies is a multidisciplinary approach [37]. Value proposition design and business modeling [36] deepen this multidisciplinary approach by bringing multiple stakeholders together to discuss values and preconditions [35].

The identification and inclusion of stakeholders for this study was furthermore based on a relevance rating according to the stakeholder salience approach [40]. This can be considered a strength as often stakeholders seem to be identified based on researchers’ intuition [46, 51] rather than how relevant stakeholders perceive themselves. Although consulting a range of different stakeholder groups was a time-consuming process, we believe this is essential to achieve a fit between technology, stakeholders, and context. We saw that each stakeholder group brought different needs to the table, so including end-users only would have limited our insights. Particularly helpful proved to be the inclusion of policy advisors and managers, as those stakeholders saw the bigger picture of a changing dementia care system and understood the global problems the technology needs to address. Ultimately, the approach taken in our study might also be useful for other cases of technology design for dementia care, and especially those cases characterized by a complex set of stakeholders with diverse agendas [33, 38].

Our study does not come without limitations. First, our results – especially those regarding preconditions for implementation – have been obtained within the context of the Dutch (dementia) care system, which might limit their generalizability to the context of other countries. However, even though differences in care systems between countries exist we believe that the topics found are sufficiently universal in nature, making them widely applicable.

Second, due to the time-consuming nature of our approach, we did not include an additional feedback loop directly after the study in order to check how far participants could find themselves in the final results. However, we plan to share the results among our participants to provide room for critical feedback.

Third, our study did not include the perspective of other stakeholders in the wider system, such as technology suppliers, to discuss preconditions for successful implementation. Although not identified as key stakeholders in our study, we recommend including their perspectives as well to prevent overlooking relevant implementation aspects. This is in line with frameworks such as NASSS [49] which shows that successful implementation of health technology is influenced by both the demand-side and supply-side.

Lastly, the grouping of key stakeholders in our study might be too simplified as each stakeholder group can basically be divided into more specific subgroups.

### Future research

We recommend developers of novel UM technology for home-based dementia care to build forward on our results by combining the multiple stakeholder value propositions and integrating them into new prototypes or re-designs. The business model canvas generated by this study can serve as a foundation for a future implementation strategy aiming to deliver the identified value propositions. Such an implementation strategy will be highly context-dependent. We therefore advise researchers of other countries to extend or refine our business model canvas with preconditions that are specific to the national dementia care system in place.

Furthermore, sustainable implementation of UM technology for home-based dementia care also depends on acceptance. It is likely that acceptance towards these technologies will vary across different (in)formal care situations [52], however, current literature does not yet provide tailored insights. Therefore, future quantitative studies on user acceptance of UM technology for home-based dementia care are warranted to better understand individual differences in acceptance.

## Conclusions

In conclusion, our study provides insight into a broader range of relevant stakeholder perspectives towards UM technology for home-based dementia care which can help to develop and implement those systems in a value-driven way. Stakeholders in this study all had their own perspective on gains and pains of UM technology, resulting into different stakeholder profiles and value maps which are, however, largely convergent. An essential design-oriented need refers to clear situation classifications to help (in)formal caregivers distinguish between urgent, non-urgent, and future care needs of PwD.

Next to that, our study adds to the limited body of work on what is needed to successfully implement UM technology in home-based dementia care. Central to a successful implementation via home care organizations seems to be inter-organizational collaboration, a shared vision on re-shaping existing care processes, integrated care ICT infrastructures, clear eligibility criteria for end-users, and flexible care reimbursement systems.

Finally, our study approach illustrates that applying well-established frameworks from the business modeling field can be useful for exploring stakeholder values and implementation preconditions in the context of technology for dementia care.

## Supplementary Information

**Additional file 1.** Stakeholder identification survey.

**Additional file 2.** Topic guide used during focus groups and interviews with key stakeholders.

## Data Availability

The datasets used and analyzed during the current study are not publicly available as participants did not explicitly state consent to this end. However, the datasets are available from the corresponding author on reasonable request.

## Abbreviations

AI: Artificial intelligence
BMC: Business Model Canvas
CeHRes: Roadmap: Centre for eHealth & Wellbeing Research Roadmap
FAT: Fairness, accountability, and transparency
ICT: Information and communication technology
LOS: Line of sight
MCI: Mild cognitive impairment
NASSS framework: Non-adoption, abandonment, scale-up, spread and sustainability framework
PwD: People/person with dementia
UM: Unobtrusive monitoring
