# Supplementary material for: How to create value with unobtrusive monitoring technology in home-based dementia care: a multimethod study among key stakeholders
Source: BMC Geriatr. 2022 Nov 30;22:921. doi: 10.1186/s12877-022-03550-1 (PMC9713088; doi:10.1186/s12877-022-03550-1)
Supplement: Supplementary file 1 — Additional file 1. Stakeholder identification survey. [file 12877_2022_3550_MOESM1_ESM.docx]

**Additional file 1: Stakeholder identification survey**

[Note: translated from the original Dutch version]

Dear madam/ sir,

We would like to involve your expertise for the next step of our project ‘’Unobtrusive sensing technologies to monitor and coach older adults with dementia’’. We have prepared a short questionnaire for this and would appreciate it if you complete it. It will take no longer than 15 minutes to fill in.

**What is this questionnaire about?**

While people with dementia would like to continue living independently at home for longer, this often puts more pressure on their (in)formal care network. To support (in)formal caregivers of people with dementia, there is growing interest in unobtrusive monitoring (UM) technology. Those are intelligent in-home sensor systems which have the potential to monitor lifestyle, health, and safety of people with dementia in a non-obtrusive (contactless) way. However, UM technologies can only reach their potential if it they meet the expectations of relevant stakeholders.

In our project we have mapped out the user perspective ((in)formal caregivers of community-dwelling people with dementia) and would like to involve a wider group of stakeholders in the next step. For this, we would like to **identify the** **most important stakeholders** with your help. Those stakeholders will be invited for focus groups and interviews about important values for UM technology and preconditions for successful implementation.

We hope for your cooperation.

With kind regards,

Christian Wrede

Dr. Annemarie Braakman-Jansen

Prof. Dr. Lisette van Gemert-Pijnen,

Dept. of Psychology, Health & Technology,

Centre for eHealth and Wellbeing Research,

University of Twente

**Instructions**

We would like to ask you to fill out the following in the table below (next page):

1. **Relevance of stakeholder**

We would like to know to what extent the attributes ''power'', ''legitimacy'', and ''urgency'' apply to various stakeholders for the development and implementation of UM technology to support the care of *community-dwelling* people with dementia. The attributes are defined as follows:

**Power:** To what extent does the stakeholder have the power to influence the development & implementation of UM technology?

**Legitimacy:** To what extent must the stakeholder be involved in the development & implementation of UM technology (e.g. based on legal, moral or contractual reasons)?

**Urgency:** To what extent do the stakeholder’s needs and wishes require immediate attention during development & implementation of UM technology?

Could you please indicate per stakeholder to what extent you assign the above attributes to the stakeholder, on a scale from 0 to 3?

0 = Not applicable at all

1 = Slightly applicable

2 = Fairly applicable

3 = Very much applicable

1. **Additions**

If you are missing stakeholders in the list, you can add those at the bottom.

| **Stakeholder** | **Relevance stakeholder** | | | |
| --- | --- | --- | --- | --- |
|  | **Power**  **(0-3)** | **Legitimacy**  **(0-3)** | **Urgency**  **(0-3)** | **N.a.** |
| **Care recipients** |  |  |  |  |
| Community-dwelling people with dementia or mild cognitive impairment |  |  |  |  |
|  |  |  |  |  |
| **Informal caregivers** |  |  |  |  |
| Informal caregivers living together with care recipient (e.g. the partner) |  |  |  |  |
| Informal caregivers caring at a distance (e.g. adult children) |  |  |  |  |
|  |  |  |  |  |
| **Home care professionals** |  |  |  |  |
| Case manager dementia |  |  |  |  |
| District nurse |  |  |  |  |
| Personal care assistant |  |  |  |  |
| Paramedics (e.g. physiotherapist, occupational therapist) |  |  |  |  |
|  |  |  |  |  |
| **Medical care specialists** |  |  |  |  |
| General practitioner |  |  |  |  |
| General practitioner assistant (POH) |  |  |  |  |
| Aged care physician |  |  |  |  |
| Clinical geriatrician |  |  |  |  |
| Neurologist |  |  |  |  |
|  | **Relevance stakeholder** | | | |
|  | **Power**  **(0-3)** | **Legitimacy**  **(0-3)** | **Urgency**  **(0-3)** | **N.a.** |
| **Aged care institutions** |  |  |  |  |
| Director/ general manager |  |  |  |  |
| Chief operating officer |  |  |  |  |
| Manager home care |  |  |  |  |
| Policy advisors care technology & innovation |  |  |  |  |
| IT administrator |  |  |  |  |
|  |  |  |  |  |
| **Municipalities** |  |  |  |  |
| Assessor care indications |  |  |  |  |
|  |  |  |  |  |
| **Health insurance companies** |  |  |  |  |
| Policy advisor aged care |  |  |  |  |
|  |  |  |  |  |
| **Social work organizations** |  |  |  |  |
| Informal care support consultant |  |  |  |  |
| Social worker |  |  |  |  |
|  |  |  |  |  |
| **Business** |  |  |  |  |
| Software companies |  |  |  |  |
|  | **Relevance stakeholder** | | | |
|  | **Power**  **(0-3)** | **Legitimacy**  **(0-3)** | **Urgency**  **(0-3)** | **N.a.** |
| **Interest organizations** |  |  |  |  |
| Patient organizations (e.g. national Alzheimer Association) |  |  |  |  |
| Informal care associations |  |  |  |  |
| Professional associations (e.g. national District Nurse Union) |  |  |  |  |
|  |  |  |  |  |
| **Science** |  |  |  |  |
| Technical engineers |  |  |  |  |
| Ethical scientists |  |  |  |  |
|  |  |  |  |  |
| **Would you like to add stakeholders?:** |  |  |  |  |
| […] |  |  |  |  |
| […] |  |  |  |  |
| […] |  |  |  |  |

Do you have any general remarks?

|  |
| --- |

Thank you very much for completing this questionnaire, we highly appreciate your help.
