# Supplementary material for: How to create value with unobtrusive monitoring technology in home-based dementia care: a multimethod study among key stakeholders
Source: BMC Geriatr. 2022 Nov 30;22:921. doi: 10.1186/s12877-022-03550-1 (PMC9713088; doi:10.1186/s12877-022-03550-1)
Supplement: Supplementary file 2 — Additional file 2. Topic guide used during focus groups and interviews with key stakeholders. [file 12877_2022_3550_MOESM2_ESM.docx]

**Additional file 2: Focus group- and interview guide**

[Note: translated from the original Dutch version]

**Topic guide used with informal caregivers**

| Topic | Questions/ instructions |
| --- | --- |
| Start | Discuss with participants:   - Introduction of researcher and overarching project - Purpose, content and structure of focus group/ interview - No right or wrong answers; participants are free to skip questions they prefer to not answer - Participants can ask questions at any time - Audio recording - Written/ digital informed consent (anonymous processing of data, data protection measures, the right to drop out at any time) - Do you have any questions before we begin? |
| Background questions | Background questionnaire (age, gender, age of loved one with dementia/ MCI, type of dementia/ MCI, year of diagnosis, relationship with loved one, housing situation) |
| Stakeholder goals | 1. What would you like to achieve in the care of your loved one with dementia/ MCI? What are your main goals as informal caregiver? 2. Which challenges do you experience in the care of your loved one with dementia/ MCI? 3. How important do you consider those goals or challenges? |
| Explanation of UM technology | See ‘’Explanation of UM technology given during focus groups and interviews’’ (page 7­­–8). |
| Gains | 1. What should be the added value of UM technology according to you? Which gains do you expect for yourself? 2. When would the use of UM technology be a success for you? 3. In front of you, you can see a number of potential gains of UM technology which emerged from a previous study among informal caregivers:  - Enabling PwD to stay longer at home - Eliminating in-person control visits - Preventing health risks - More reassurance - Providing others with an objective insight into the situation of PwD   Which of those gains do you consider most and least important and why?  Are there any gains missing in this list? If so, what are those? |
| Pains | 1. What are possible pains or disadvantages of using UM technology according to you? 2. What are possible risks or undesirable results that need to be avoided? 3. In front of you, you can see a number of potential pains of UM technology which emerged from a previous study among informal caregivers:  - Information overload - Too privacy-invasive - Risk of reduced human contact   Which of those pains do you consider most and least important and why?  Are there any pains missing in this list? If so, what are those? |
| Gain creators | In this step we are interested in what is needed from UM technology to realize the gains we just discussed. Please feel free to imagine anything, regardless of whether it would be technically possible at the moment.   1. Which of the gains we discussed should be translated into concrete characteristics of UM technology? What would those characteristics be? (‘’The technology must …’’) 2. Which characteristics or features of UM technology are needed to support you in the best possible way? |
| Pain relievers | We are also curious if you have ideas about what is needed from UM technology to relieve the pains we discussed. Please feel free to imagine anything, regardless of whether it would be technically possible at the moment.   1. Which of the pains we discussed should be translated into concrete characteristics of UM technology? What would those characteristics be? (‘’The technology must …’’) 2. How should we take into account certain pains during the development of UM technology? |
| End | Are there any aspects that we have not yet discussed in the previous sections that you would like to share?  Thank you very much for your time and valuable help. |

**Topic guide used with home care professionals**

| Topic | Questions/ instructions |
| --- | --- |
| Start | Discuss with participants:   - Introduction of researcher and overarching project - Purpose, content and structure of focus group/ interview - No right or wrong answers; participants are free to skip questions they prefer to not answer - Participants can ask questions at any time - Audio recording - Written/ digital informed consent (anonymous processing of data, data protection measures, the right to drop out at any time) - Do you have any questions before we begin? |
| Background questions | Background questionnaire (age, gender, function, number of years of work experience in current home care profession, care contact hours per week provided to community-dwelling clients with dementia/ MCI) |
| Stakeholder goals | 1. What would you like to achieve in the care of your community-dwelling clients with dementia/ MCI? What are your main goals as home care professional working with this specific group? 2. Which challenges do you experience in the care of your community-dwelling clients with dementia/ MCI? 3. How important do you consider those goals or challenges? |
| Explanation of UM technology | See ‘’Explanation of UM technology given during focus groups and interviews’’ (page 7­­–8). |
| Gains | 1. What should be the added value of UM technology according to you? Which gains do you expect for yourself? 2. When would the use of UM technology be a success for you? 3. In front of you, you can see a number of potential gains of UM technology which emerged from a previous study among home care professionals:  - Enabling PwD to stay longer at home - Eliminating in-person control visits - Preventing health risks - Personalized care - Objective information   Which of those gains do you consider most and least important and why?  Are there any gains missing in this list? If so, what are those? |
| Pains | 1. What are possible pains or disadvantages of using UM technology according to you? 2. What are possible risks or undesirable results that need to be avoided? 3. In front of you, you can see a number of potential pains of UM technology which emerged from a previous study among home care professionals:  - Information overload - Too privacy-invasive - Risk of undermining the formal caregiver’s professional view   Which of those pains do you consider most and least important and why?  Are there any pains missing in this list? If so, what are those? |
| Gain creators | In this step we are interested in what is needed from UM technology to realize the gains we just discussed. Please feel free to imagine anything, regardless of whether it would be technically possible at the moment.   1. Which of the gains we discussed should be translated into concrete characteristics of UM technology? What would those characteristics be? (‘’The technology must …’’) 2. Which characteristics or features of UM technology are needed to support you in the best possible way? |
| Pain relievers | We are also curious if you have ideas about what is needed from UM technology to relieve the pains we discussed. Please feel free to imagine anything, regardless of whether it would be technically possible at the moment.   1. Which of the pains we discussed should be translated into concrete characteristics of UM technology? What would those characteristics be? (‘’The technology must …’’) 2. How should we take into account certain pains during the development of UM technology? |
| Preconditions for successful implementation | **General**   1. When is the implementation of UM technology in home-based dementia care a success for you? 2. Do you have lessons learned from previous implementation trajectories that could also apply to UM technology?   **Needed infrastructure (key partners, key activities, key resources)**   1. Which partners and resources do you need to successfully implement UM technology in home-based dementia care? 2. Which (technical) infrastructure is needed? 3. Which implementation activities are essential? 4. How can UM technology best be embedded into the existing work structure? What kind of changes are needed?   **End-users (end-user segments, channels, end-user relationships)**   1. Who exactly should be offered UM technology in home-based dementia care? 2. What is the best way to offer UM technology?   **Costs & revenues**   1. Which financial preconditions need to be taken into account for successful implementation of UM technology in home-based dementia care? |
| End | Are there any aspects that we have not yet discussed in the previous sections that you would like to share?  Thank you very much for your time and valuable help. |

**Topic guide used with people with dementia/ MCI**

| Topic | Questions/ instructions |
| --- | --- |
| Start | Discuss with participants:   - Introduction of researcher and overarching project - Purpose, content and structure of focus group/ interview - No right or wrong answers; participants are free to skip questions they prefer to not answer - Participants can ask questions at any time - Audio recording - Written/ digital informed consent (anonymous processing of data, data protection measures, the right to drop out at any time) - Do you have any questions before we begin? |
| Background questions | Background questions (age, number of children (if any), housing situation, assistance of (in)formal caregivers) |
| Stakeholder goals | 1. How do you imagine your preferred living or care situation in the coming time/ years? Do you have a specific goal? How important is this goal to you? 2. Are there challenges in everyday life that you run into? How urgent are those challenges according to you? |
| Explanation of UM technology | See ‘’Explanation of UM technology given during focus groups and interviews’’ (page 7­­–8). |
| Gains | 1. What should be the added value of UM technology according to you? What do you think are the benefits? 2. In which way do you think that UM technology might help you and your caregivers (if at all)? 3. How do you think the use of UM technology might influence your care? |
| Pains | 1. What are possible disadvantages of using UM technology according to you? 2. Why would you decide to not use UM technology at home? 3. What are possible risks or undesirable results for yourself that need to be avoided? When would UM technology become undesirable for you? |
| Gain creators | In this step we are interested in what is needed from UM technology to realize the benefits we just discussed. Please feel free to imagine anything, regardless of whether it would be technically possible at the moment.   1. Which of the benefits we discussed should be translated into concrete characteristics of UM technology? What would those characteristics be? (‘’The technology must …’’) 2. Which preconditions for UM technology are needed so that it would be acceptable for you? |
| Pain relievers | We are also curious if you have ideas about what is needed from UM technology to relieve the disadvantages we discussed. Please feel free to imagine anything, regardless of whether it would be technically possible at the moment.   1. Which of the disadvantages we discussed should be translated into concrete characteristics of UM technology? What would those characteristics be? (‘’The technology must …’’) 2. How should we take into account certain disadvantages during the development of UM technology? |
| End | Are there any aspects that we have not yet discussed in the previous sections that you would like to share?  Thank you very much for your time and valuable help. |

**Topic guide used with directors, managers, and policy advisors**

| Topic | Questions/ instructions |
| --- | --- |
| Start | Discuss with participants:   - Introduction of researcher and overarching project - Purpose, content and structure of focus group/ interview - No right or wrong answers; participants are free to skip questions they prefer to not answer - Participants can ask questions at any time - Audio recording - Written/ digital informed consent (anonymous processing of data, data protection measures, the right to drop out at any time) - Do you have any questions before we begin? |
| Background questions | Background questionnaire (age, current function, number of years of work experience in current function) |
| Stakeholder goals | 1. What would you like to achieve for the care of community-dwelling people with dementia/ MCI? What are the main goals from your perspective? 2. How important do you consider those goals? |
| Explanation of UM technology | See ‘’Explanation of UM technology given during focus groups and interviews’’ (page 7­­–8). |
| Gains | 1. What should be the added value of UM technology for home-based dementia care? 2. When would the use of UM technology be a success for your organization? Which outcomes would you like to generate with the use of UM technology in home-based dementia care? 3. In front of you, you can see a number of potential gains of UM technology which emerged from a previous study among informal caregivers and home care professionals:  - Enabling PwD to stay longer at home - Preventing health risks - Personalized care - Objective information - More reassurance - Eliminating in-person control visits   Which of those gains do you consider most and least important and why?  Are there any gains missing in this list? If so, what are those? |
| Pains | 1. What are possible pains or disadvantages of using UM technology in home-based dementia care? 2. What are possible risks or undesirable results that need to be avoided? 3. In front of you, you can see a number of potential pains of UM technology which emerged from a previous study among informal caregivers and home care professionals:  - Information overload - Too privacy-invasive - Risk of reduced human contact - Undermining the formal caregiver’s professional view   Which of those pains do you consider most and least important and why?  Are there any pains missing in this list? If so, what are those? |
| Gain creators | In this step we are interested in what is needed from UM technology to realize the gains we just discussed. Please feel free to imagine anything, regardless of whether it would be technically possible at the moment.   1. Which of the gains we discussed should be translated into concrete characteristics of UM technology? What would those characteristics be? (‘’The technology must …’’) |
| Pain relievers | We are also curious if you have ideas about what is needed from UM technology to relieve the pains we discussed. Please feel free to imagine anything, regardless of whether it would be technically possible at the moment.   1. Which of the pains we discussed should be translated into concrete characteristics of UM technology? What would those characteristics be? (‘’The technology must …’’) 2. How should we take into account certain pains during the development of UM technology? |
| Preconditions for successful implementation | **General**   1. When is the implementation of UM technology in home-based dementia care a success for you? 2. Do you have lessons learned from previous implementation trajectories that could also apply to UM technology?   **Needed infrastructure (key partners, key activities, key resources)**   1. Which partners and resources do you need to successfully implement UM technology in home-based dementia care? 2. Which (technical) infrastructure is needed? 3. Which implementation activities are essential? 4. How can UM technology best be embedded into the existing work structure? What kind of changes are needed?   **End-users (end-user segments, channels, end-user relationships)**   1. Who exactly should be offered UM technology in home-based dementia care? 2. What is the best way to offer UM technology?   **Costs & revenues**   1. Which financial preconditions need to be taken into account for successful implementation of UM technology in home-based dementia care? |
| End | Are there any aspects that we have not yet discussed in the previous sections that you would like to share?  Thank you very much for your time and valuable help. |

**Explanation of UM technology given during focus groups and interviews**

*Imagine the following:*

*The monitoring system provides information about the daily living pattern, behavior or mood of a resident, especially in situations where an informal or professional caregiver is not around. The information from the system can be visualized on a digital platform and can provide insight into the situation of a resident. The system uses artificial intelligence which means it is a self-learning system which can learn to recognize daily living patterns over time. In this way, also subtle changes can be detected. Alarms are possible in certain situations. For instance, when the resident shows no activity for longer time, or any other situation in which in an alarm is desired. Furthermore, the monitoring system works contactless and wirelessly. This means that the resident does not have to wear any devices on or close to the body. The monitoring system can be placed in a central location of the room/ appartment to monitor the situation of a resident remotely, even through the wall. Within the care network, three potential recipients of the monitoring information exist: 1) informal caregiver(s) of the resident, 2) healthcare professional(s) involved in the resident’s care, and 3) the resident him/her self.*


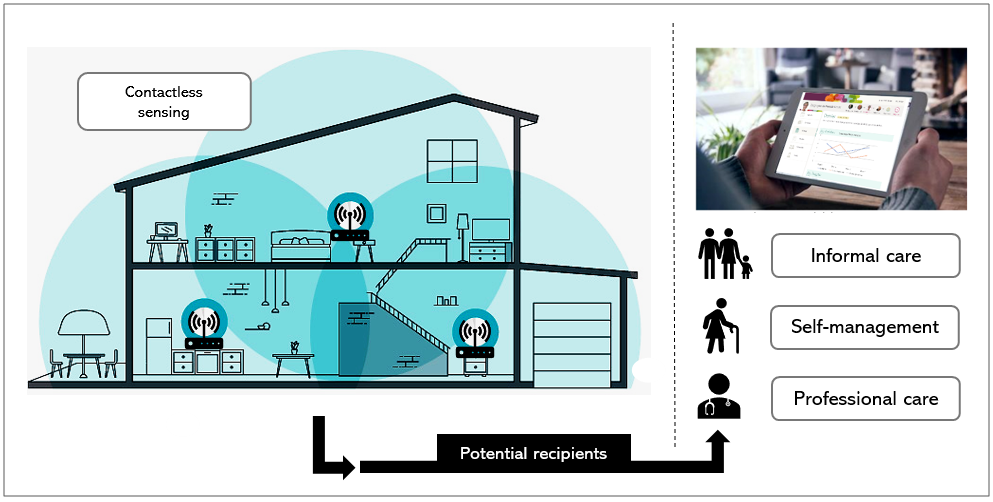


Slide shown to demonstrate the general concept of UM technology. Note: Permission to use

parts of the figure was granted by [tp-link.com](https://www.tp-link.com/nl/).

**
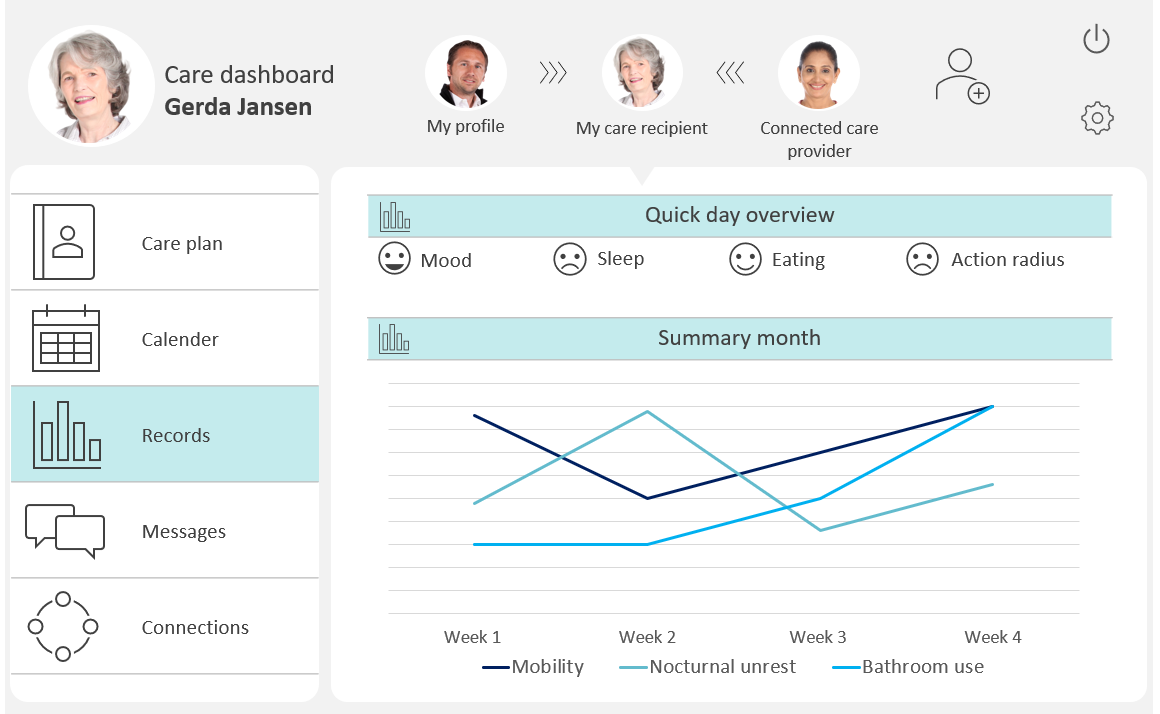
**

Slide shown to illustrate an example of outgoing monitoring information, visualized on a digital

care collaboration platform.
